# A carbapenem-focused antimicrobial stewardship programme implemented during the COVID-19 pandemic in a setting of high endemicity for multidrug-resistant Gram-negative bacteria

**DOI:** 10.1093/jac/dkad035

**Published:** 2023-02-15

**Authors:** Nikolaos Spernovasilis, Evangelos I Kritsotakis, Anna Mathioudaki, Alexandra Vouidaski, Christos Spanias, Maria Petrodaskalaki, Petros Ioannou, Georgios Chamilos, Diamantis P Kofteridis

**Affiliations:** School of Medicine, University of Crete, Heraklion, Greece; Department of Internal Medicine and Infectious Diseases, University Hospital of Heraklion, Heraklion, Greece; School of Medicine, University of Crete, Heraklion, Greece; Laboratory of Biostatistics, School of Medicine, University of Crete, Heraklion, Greece; Department of Internal Medicine and Infectious Diseases, University Hospital of Heraklion, Heraklion, Greece; Department of Internal Medicine and Infectious Diseases, University Hospital of Heraklion, Heraklion, Greece; Department of Pharmacy, University Hospital of Heraklion, Heraklion, Greece; Department of Quality & Research, University Hospital of Heraklion, Heraklion, Greece; School of Medicine, University of Crete, Heraklion, Greece; Department of Internal Medicine and Infectious Diseases, University Hospital of Heraklion, Heraklion, Greece; School of Medicine, University of Crete, Heraklion, Greece; Department of Clinical Microbiology, University Hospital of Heraklion, Heraklion, Greece; School of Medicine, University of Crete, Heraklion, Greece; Department of Internal Medicine and Infectious Diseases, University Hospital of Heraklion, Heraklion, Greece

## Abstract

**Background:**

Greece is among the countries characterized by high rates of antimicrobial resistance and high consumption of antibiotics, including carbapenems.

**Objectives:**

To measure the impact of a carbapenem-focused antimicrobial stewardship programme (ASP) on the antibiotic consumption and patient outcomes in a Greek tertiary hospital during the COVID-19 pandemic.

**Methods:**

A quasi-experimental, before–after study, comparing a 12 month pre-intervention period with a 12 month intervention period in which a carbapenem-focused ASP was implemented.

**Results:**

A total of 1268 patients were enrolled. The proportion of admitted patients who received carbapenems decreased from 4.1% (842 of 20 629) to 2.3% (426 of 18 245) (−1.8%; *P* < 0.001). A decrease of −4.9 DDD/100 patient-days (PD) (95% CI −7.3 to −2.6; *P* = 0.007) in carbapenem use and an increase in the use of piperacillin/tazobactam [+2.1 DDD/100 PD (95% CI 1.0–3.3; *P* = 0.010)] were observed. Thirty-day mortality following initiation of carbapenem treatment and all-cause in-hospital mortality remained unaltered after ASP implementation. In contrast, length of hospital stay increased (median 17.0 versus 19.0 days; *P* < 0.001), while the risk of infection-related readmission within 30 days of hospital discharge decreased (24.6% versus 16.8%; *P* = 0.007). In the post-implementation period, acceptance of the ASP intervention was associated with lower daily hazard of in-hospital death [cause-specific HR (csHR) 0.49; 95% CI 0.30–0.80], lower odds of 30 day mortality (OR 0.36; 95% CI 0.18–0.70) and higher rate of treatment success (csHR 2.45; 95% CI 1.59–3.77).

**Conclusions:**

Implementing and maintaining a carbapenem-focused ASP is feasible, effective and safe in settings with high rates of antimicrobial resistance, even during the COVID-19 pandemic.

## Introduction

Carbapenems are important elements of the antibiotic armamentarium, with established efficacy against most infections caused by MDR Gram-negative bacteria (GNB). Their efficacy is mainly due to (i) their stability against most β-lactamases, including the AmpCs and the ESBLs, and (ii) the broad spectrum of their activity.^1^ In addition, carbapenems have a better safety profile compared with other last-line antibiotics, such as polymyxins.^1^ However, inappropriate use of these broad-spectrum β-lactam antibiotics aggravates the problem of antimicrobial resistance (AMR) by promoting the emergence of XDR and pandrug-resistant Gram-negative nosocomial pathogens through the induction of selective pressure.^2,3^

Over the last decade, Greece has ranked among the countries with the highest consumption of antibiotics in Europe, including carbapenems and other broad-spectrum antibiotics, both in community and hospital settings.^4^ In parallel, the country’s AMR rates, including carbapenem-resistant GNB, have been extremely high, and this is also the case in some other European countries.^5^ The observed infections due to MDR-GNB in these countries pose a significant challenge for clinicians and a major threat for healthcare systems due to their high attributable mortality and hospital costs.^6^

The threat of AMR may become more evident in the years to come, in part due to the high and often inappropriate use of antimicrobials during the coronavirus disease 2019 (COVID-19) pandemic.^7^ Especially in the early phases of the pandemic, antimicrobials were widely used as repurposed drugs and as empirical coverage of coinfections and superinfections in COVID-19 patients.^8^ However, no reliable scientific evidence supports the use of antibiotics, antiretrovirals and antiparasitics as repurposed drugs against severe acute respiratory syndrome coronavirus 2.^9^ In addition, almost three-quarters of COVID-19 patients received antibiotics during the first months of the pandemic, but only a minority of them had documented bacterial coinfection or superinfection.^10^ This observed overprescription included carbapenems in several studies of hospitalized patients, especially in ICU.^11–14^

Accordingly, a pivotal target of antimicrobial stewardship programmes (ASPs) is to decrease unnecessary administration of carbapenems. To optimize carbapenem prescription in our hospital, a setting with high rates of MDR-GNB, a carbapenem-focused ASP was implemented during the first year of the COVID-19 pandemic. The aim of our study was to examine the impact of this ASP on the consumption of antibiotics used to treat MDR-GNB and on patient safety and outcomes.

## Materials and methods

### Study design, setting and population

This retrospective-prospective, before–after, quasi-experimental study was conducted at a 770 bed tertiary university hospital that covers all surgical and medical specialties, including cardiac surgery, neurosurgery, surgical oncology, rheumatology, oncology, haematology and ICU. Before January 2020 there was no formal ASP implemented in the study site. The pre-implementation period from January 2019 to December 2019 was retrospectively evaluated and was compared with the intervention period of January 2020 to December 2020.

The study enrolled all patients ≥16 years of age who received carbapenems (i.e. meropenem, imipenem or ertapenem) for at least 24 h during the 24 month study period. Those who received more than one course of carbapenems during each of the study subperiods were only included once in the corresponding subperiod analysis, the first time they received the carbapenem antibiotic. Patients who died within 24 h of carbapenem administration or had been transferred from another hospital and had received carbapenem therapy during their hospitalization at that hospital were excluded.

The study was approved by the hospital’s Review Board. The need for the patient’s informed consent was waived because the study represented customary medical practice and the ASP complied with national medical guidelines and legislation for the control of AMR in Greece.

### Intervention

Starting 1 January 2020, a multifaceted ASP was implemented to optimize the prescription of carbapenems with regard to indication, dosage and duration of administration. Whenever appropriate, the intervention promoted recommendation for judicious use of carbapenem-sparing antibiotics. The ASP team comprised of an infectious disease (ID) specialist, an ID fellow, a microbiologist and a pharmacist. The programme was based on the strategy of prospective audit and feedback to prescribers and was supplemented by parallel case-based educational sessions, meetings and presentations on proper use of antibiotics.

The ID specialist and the ID fellow were alerted by the pharmacy upon prescription order for a carbapenem and provided unsolicited in-person (‘handshake’) consultation within 72 h for all adult patients receiving a carbapenem antibiotic. Further ID consultation service was available 24/7 through telephone or in person upon request by the treating doctors. Unsolicited follow-up bedside ID consultation was provided daily or every other day for patients whose treating physicians had accepted the intervention. After examining each eligible patient and reviewing their medical record, the ID specialist or the ID fellow discussed with the prescribers whether continuing carbapenems or using non-carbapenem antibiotics for empirical treatment would be appropriate. Whenever relevant microbiological data were available, the options of targeted de-escalation to narrow-spectrum antibiotics or targeted escalation to ceftazidime/avibactam, tigecycline or colistin were considered. Of note, and only when susceptibility data were available, ceftolozane/tazobactam was used as a carbapenem-sparing treatment option while ceftazidime/avibactam was used only for the targeted treatment of carbapenem-resistant GNB. Treating physicians were not obligated to comply with ASP team’s recommendations.

### Variables

Antibiotic consumption data per calendar quarter for carbapenems, piperacillin/tazobactam, ceftolozane/tazobactam, ceftazidime/avibactam, tigecycline and colistin were retrieved from the hospital pharmacy records and were expressed as DDD per 100 patient-days (PD).

Patient demographics, clinical characteristics, length of hospital stay, and outcomes were retrospectively reviewed during the pre-intervention period and prospectively collected during the intervention period. Outcome endpoints included inpatient death, death within 30 days of carbapenem initiation (including post-discharge cases) and infection-related readmission within 30 days of hospital discharge. Outcome during or at the end of the antibiotic treatment could be assessed only for the post-implementation cohort and was classified as death, new/recurrent infection or favourable outcome. For every patient in the post-implementation cohort, it was recorded whether the treating physician accepted the ASP recommendation or not.

### Statistical analysis

The effect of the ASP implementation on hospital antibiotic use was assessed using interrupted time series analyses. A segmented Poisson regression model was employed to examine the extent to which the ASP was associated with an immediate level change and/or a gradual trend change of the monthly numbers of carbapenem-treated patients. In this model, the series of monthly counts of carbapenem-treated patients formed the dependent variable. Independent variables were the time elapsed since the start of the study, the ASP implementation indicator (post- versus pre-ASP) and the time after the intervention. The monthly series of hospital admissions (log transformed) was used as an offset variable to convert the outcome into a rate that accounts for variation in the hospital population size over time. Two pairs of sine–cosine Fourier functions of time were included to capture seasonality. The model coefficients were estimated using the maximum-likelihood method. Residual autocorrelation was ruled out by examining autocorrelation graphs. Sensitivity analyses were conducted by using the monthly numbers of hospitalized patients and PD as alternative denominators for the treatment rate, and by inflating the standard errors by the scaled Pearson chi-squared statistics to adjust for the possibility of overdispersion.

In addition, we examined the temporal trends in the consumption of carbapenems and other selected antibiotics with activity against MDR-GNB by using quarterly hospital data. A level-change linear regression model for interrupted time series was used for this purpose. Stratification per quarter was employed to adjust for seasonality. The model was estimated using the ordinary least squares method, and Newey–West standard errors were used to account for autocorrelation.

The impact on patient outcomes was assessed on an ITT principle by comparing all carbapenem-treated patients between the pre-implementation period and the ASP intervention period. Pearson's chi-squared test was used to assess between-group differences in overall proportions of in-hospital mortality, total mortality within 30 days of initiation of carbapenem treatment, and infection-related readmission within 30 days of hospital discharge. The Wilcoxon rank-sum test was used to assess between-group differences in length of hospital stay. Multivariable Cox regression was employed to obtain cause-specific HRs (csHRs) for in-hospital death and discharge alive, adjusting for differences in baseline covariates. The time origin was set to hospital admission. Discharge alive from the hospital was treated as a competing event to in-hospital death. In this analysis, a low csHR for discharge alive reflects a low daily rate of discharge resulting in prolonged hospital stay. Multivariable logistic regression was employed to estimate OR for total mortality within 30 days of initiation of carbapenem treatment and OR for infection-related readmission within 30 days of hospital discharge, correcting for differences in baseline covariates. All models adjusted for patient sex, age, ward of hospitalization, and history of previous hospitalization.

A series of sensitivity analyses were performed to assess the likely clinical impact of the ASP intervention under different conditions. On a modified ITT analysis, we compared the pre-implementation cohort to the post-implementation cohort, excluding patients for whom the intervention was not accepted. On per-protocol analysis, we compared patients who did not receive the intervention in either the pre- or the post-implementation period with those who received the intervention. Finally, restricting the analysis within the post-implementation period, we compared patients for whom the intervention was accepted with patients for whom the intervention was not. In the latter analysis, we additionally compared the clinical outcome at the end of therapy.

None of the study variables had missing data. Statistical significance was considered at the usual *P* < 0.05 threshold. Data processing and statistical modelling were performed using Stata version 17 (Stata Corp., College Station, TX, USA).

## Results

### Antibiotic consumption

**Figure 1.**
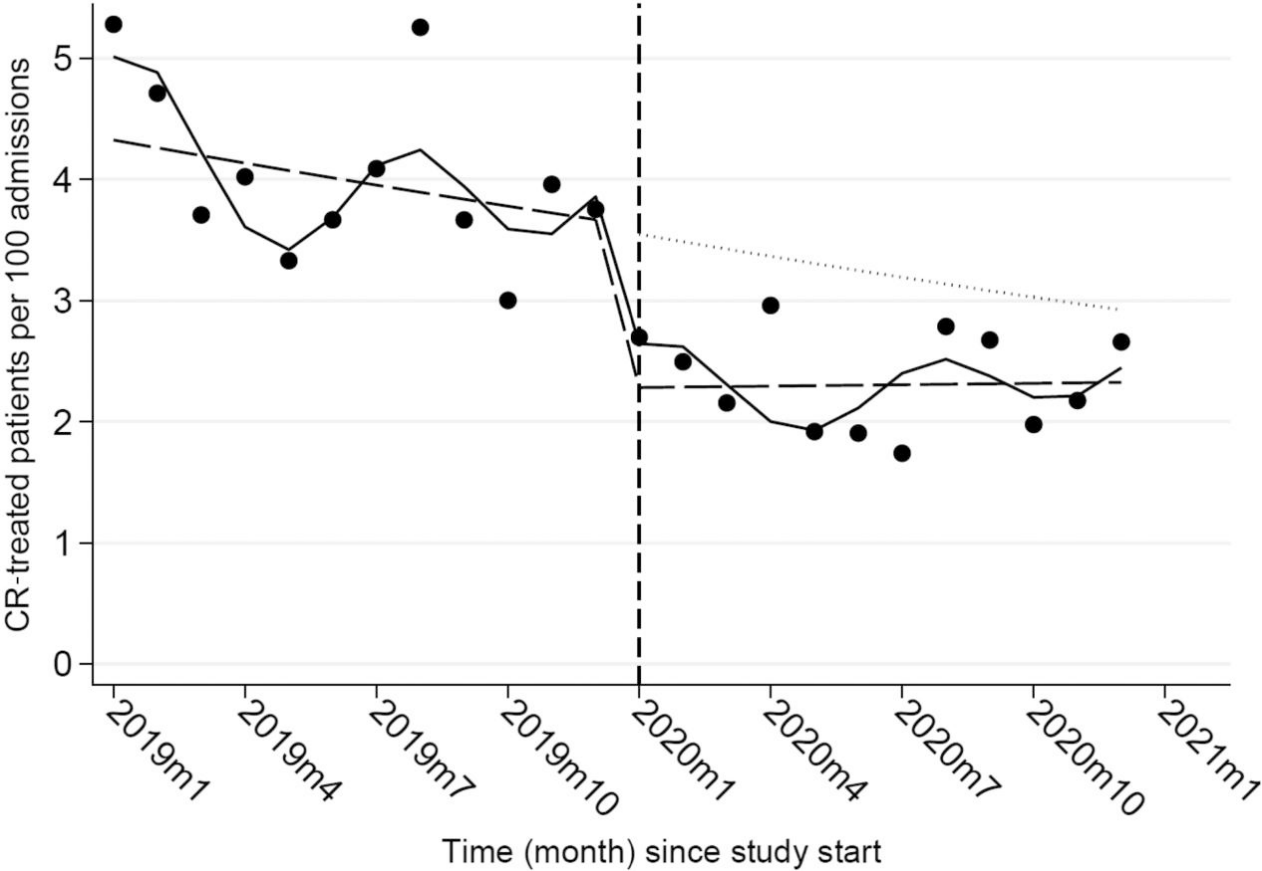
Monthly rates of carbapenem (CR)-treated patients per 100 hospital admissions, pre- and post-implementation of the ASP. Dots show observed rates, the solid line shows predicted rates from Poisson regression model adjusted for seasonality and overdispersion, the dashed line shows the deseasonalized trend, the dotted line shows the counterfactual scenario assuming the intervention was not implemented, and the vertical dashed line shows the time of the beginning of the intervention.

In all, 1329 carbapenem courses were administered to 1268 patients during the 2 year study period, 55 of whom received more than one course of carbapenems in any of the two study subperiods. After the ASP implementation, the proportion of admitted patients who received carbapenem treatment decreased significantly, from 4.1% (842 of 20 629) to 2.3% (426 of 18 245) (−1.8%; *P* < 0.001). The interrupted time series analysis confirmed that the implementation of the carbapenem-focused ASP was associated with an overall level reduction in the rate of carbapenem treatments per 100 hospital admissions [incidence rate ratio (IRR) 0.63; 95% CI 0.50–0.80; *P* < 0.001], while no substantial trend change occurred after the ASP implementation (IRR 1.02; 95% CI 1.00–1.04; *P* = 0.117; Figure 1). Sensitivity analyses confirmed that the estimated level change in the rate of carbapenem-treated patients was robust against different statistical modelling specifications and rate denominators (Table 1).

**Table 1.** Sensitivity analyses of estimating the effect of the ASP implementation on the rate of carbapenem prescription for various types of model specifications

Outcome being modelled	Poisson model specifications	IRR	95% CI	*P* value
Monthly rate of carbapenem-treated patients, per 100 hospital admissions	Unadjusted	0.70	0.56–0.87	0.002
	Adjusted for seasonality	0.63	0.50–0.80	<0.001
	Adjusted for seasonality, overdispersion	0.63	0.39–1.01	0.056
Monthly rate of carbapenem-treated patients, per 100 inpatients	Unadjusted	0.71	0.57–0.88	0.002
	Adjusted for seasonality	0.69	0.55–0.86	0.001
	Adjusted for seasonality, overdispersion	0.69	0.44–1.08	0.103
Monthly rate of carbapenem-treated patients, per 1000 PD	Unadjusted	0.68	0.55–0.85	0.001
	Adjusted for seasonality	0.71	0.57–0.88	0.002
	Adjusted for seasonality, overdispersion	0.71	0.46–1.08	0.107

**Figure 2.**
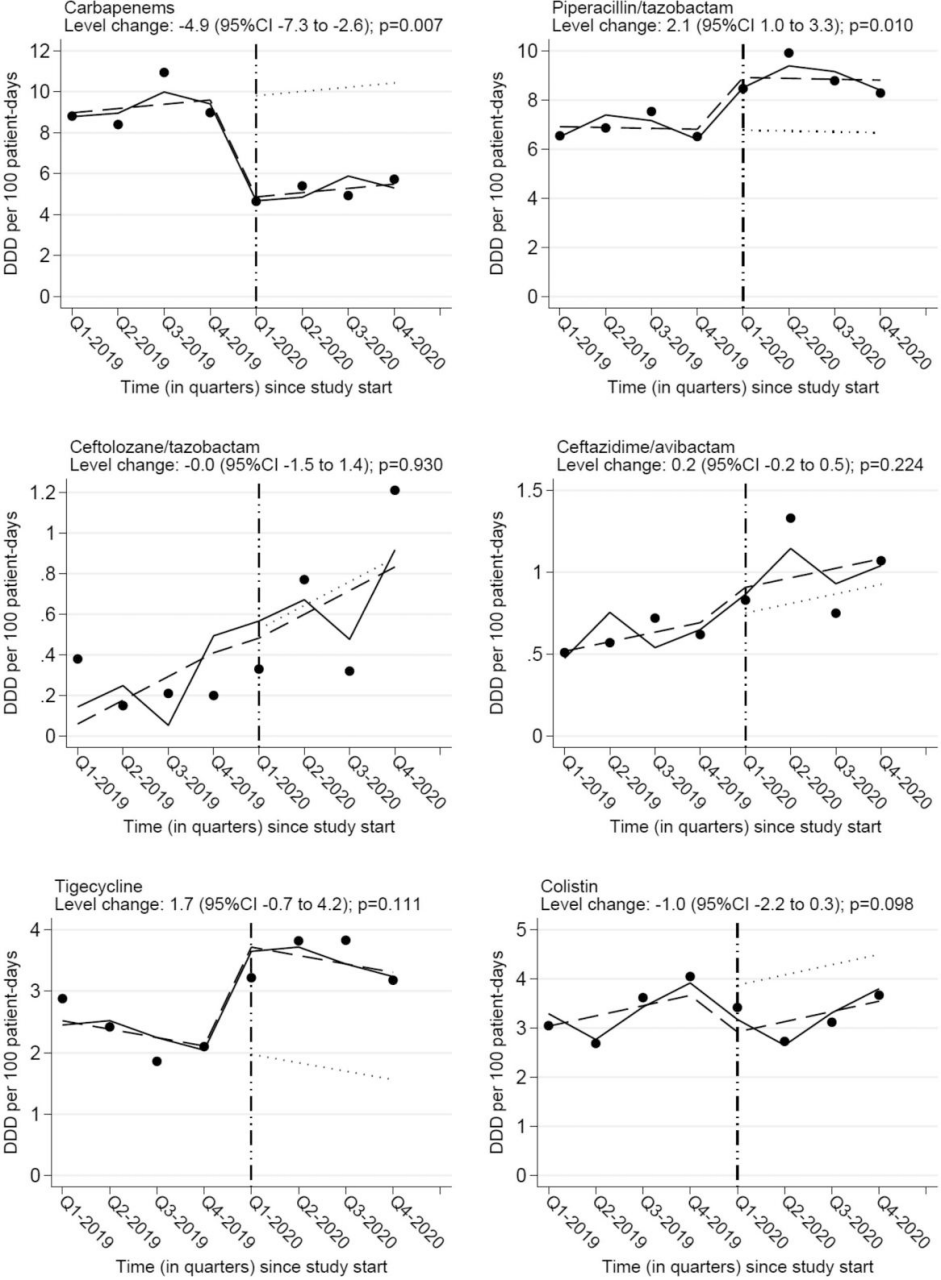
Interrupted time series graphs showing level changes in the consumption of carbapenems and other selected antibiotics with activity against MDR-GNB following the ASP implementation. The dots correspond to quarterly antibiotic consumption rates measured in DDD per 100 PD. The solid line shows the predicted rates from a segmented linear regression model adjusted for seasonality and autocorrelation. The dashed line shows the deseasonalized trend. The dotted line corresponds to the counterfactual scenario assuming the intervention was not implemented.

Analysis of quarterly data on hospital consumption of carbapenems showed that the ASP was associated with a decrease of −4.9 DDD/100 PD (95%CI −7.3 to −2.6; *P* = 0.007). A concurrent increase in the consumption of piperacillin/tazobactam was noted [+2.1 DDD/100 PD (95% CI 1.0–3.3; *P* = 0.010)]. There was also a non-statistically significant increase of tigecycline consumption and decrease of colistin consumption. The consumption of ceftolozane/tazobactam and ceftazidime/avibactam remained largely unaffected (Table 2 and Figure 2).

**Table 2.** Quarterly hospital consumption of carbapenems and other selected antibiotics with activity against MDR-GNB and level changes due to the ASP implementation (measured in DDD per 100 PD)

Antibiotic group or agent	2019 (pre-intervention year)				2020 (intervention year)				After–before level change		
	Q1	Q2	Q3	Q4	Q1	Q2	Q3	Q4	Estimate	95% CI	*P* value
Carbapenems	8.81	8.40	10.94	8.98	4.65	5.40	4.93	5.73	−4.9	−7.3 to −2.6	0.007
ȃMeropenem	8.67	8.33	10.80	8.91	4.56	5.20	4.65	5.44	−5.0	−7.3 to −2.6	0.007
ȃImipenem	0.02	0.00	0.02	0.01	0.02	0.00	0.04	0.00	0.0	−0.0 to 0.1	0.145
ȃErtapenem	0.12	0.07	0.12	0.06	0.07	0.20	0.24	0.29	0.0	−0.4 to 0.4	0.995
Piperacillin/tazobactam	6.54	6.86	7.53	6.51	8.46	9.91	8.78	8.28	2.1	1.0–3.3	0.010
Ceftolozane/tazobactam	0.38	0.15	0.21	0.20	0.33	0.77	0.32	1.21	0.0	−1.5 to 1.4	0.930
Ceftazidime/avibactam	0.51	0.57	0.72	0.62	0.83	1.33	0.75	1.07	0.2	−0.2 to 0.5	0.224
Tigecycline	2.88	2.42	1.86	2.10	3.22	3.82	3.83	3.18	1.7	−0.7 to 4.2	0.111
Colistin	3.05	2.69	3.62	4.05	3.42	2.73	3.12	3.67	−1.0	−2.2 to 0.3	0.098

Level changes were estimated by segmented linear regression adjusting for seasonality and autocorrelation. Q, quarter.

### Clinical outcomes

Demographic and clinical characteristics of the patients before and after ASP implementation did not differ significantly, as summarized in Table 3. Mortality within 30 days of initiation of carbapenem treatment (22.4% versus 23.1%; *P* = 0.798) and all-cause in-hospital mortality (23.6% versus 28.4%; *P* = 0.065) remained unaltered after ASP implementation. In contrast, length of hospital stay increased (median 17.0 versus 19.0 days; *P* < 0.001), while the risk of infection-related readmission within 30 days of hospital discharge decreased (24.6% versus 16.8%; *P* = 0.007). Multivariable regression analyses showed similar effect sizes after adjustment for baseline differences in patient sex, age, ward of hospitalization, and history of previous hospitalization (Tables S1–S19, available as Supplementary data at *JAC* Online). The results of sensitivity analyses in Table 4 confirmed that the effects of the ASP on patient outcomes were consistent under different assumed conditions.

**Table 3.** Comparison of patient characteristics and outcomes on an ITT principle (pre- versus post-implementation of the ASP) and per acceptance of the antimicrobial stewardship intervention

	ITT analysis			Acceptance analysis (post-implementation cohort)		
Variables	Pre-implementation cohort (*n* = 842)	Post-implementation cohort (*n* = 426)	*P* value	Intervention not accepted (*n* = 46)	Intervention accepted (*n* = 380)	*P* value
Male sex, *n* (%)	540 (64.1)	266 (62.4)	0.554	33 (71.7)	233 (61.3)	0.168
Age, years, median (IQR)	68.0 (56.0–78.0)	69.0 (58.0–79.0)	0.422	73.0 (64.0–84.0)	69.0 (56.0–78.0)	0.048
Ward of hospitalization, *n* (%)			0.591			0.200
ȃIntensive care	92 (10.9)	54 (12.7)		2 (4.4)	52 (13.7)	
ȃMedicine	529 (62.8)	263 (61.7)		29 (63.0)	234 (61.5)	
ȃSurgery	217 (25.8)	105 (24.6)		15 (32.6)	90 (23.7)	
ȃOther	4 (0.5)	4 (0.9)		0 (0.0)	4 (1.1)	
Previous hospitalization, *n* (%)	493 (58.6)	236 (55.4)	0.284	20 (43.5)	216 (56.8)	0.085
CCI, median (IQR)	NA	2.0 (1.0–4.0)	NA	3.0 (2.0–6.0)	2.0 (1.0–4.0)	0.104
Length of hospital stay (days) before carbapenem therapy, median (IQR)	5.0 (1.0–11.0)	5.0 (1.0–12.0)	0.272	6.0 (3.0–14.0)	4.5 (1.0–12.0)	0.279
Length of hospital stay (days), median (IQR)	17.0 (9.0–31.0)	19.0 (12.0–37.0)	<0.001	17.0 (10.0–33.0)	20.0 (12.0–38.0)	0.497
In-hospital any-cause death, *n* (%)	199 (23.6)	121 (28.4)	0.065	21 (45.7)	100 (26.3)	0.006
Death within 30 days of carbapenem initiation, *n* (%)	187 (22.4)	98 (23.1)	0.798	18 (39.1)	80 (21.1)	0.006
Infection-related readmission within 30 days of discharge alive, *n* (%)	153 (24.6)	51 (16.8)	0.007	6 (24.0)	45 (16.1)	0.313
Treatment outcome, *n* (%)						<0.001
ȃDeath				15 (32.6)	62 (16.3)	
ȃNew or recurrent infection				8 (17.4)	9 (2.4)	
ȃSuccess				23 (50.0)	309 (81.3)	

NA, not available; CCI, Charlson comorbidity index.

**Table 4.** Results of multivariable Cox proportional hazards regression and logistic regression quantifying the effects of the ASP on patient outcomes under different conditions (sensitivity analyses)

	ITT analysis^a^		Modified ITT analysis^b^		Per-protocol analysis^c^		Per-acceptance analysis^d^	
Clinical outcomes and effect measure	ES (95% CI)	*P* value	ES (95% CI)	*P* value	ES (95% CI)	*P* value	ES (95% CI)	*P* value
In-hospital death, csHR	0.99 (0.79–1.24)	0.922	0.91 (0.71–1.16)	0.450	0.87 (0.69–1.11)	0.256	0.49 (0.30–0.80)	0.004
Discharge alive, csHR	0.80 (0.70–0.92)	0.002	0.83 (0.72–0.95)	0.009	0.85 (0.74–0.98)	0.022	1.32 (0.87–2.00)	0.187
30 day death, OR^e^	1.26 (0.95–1.67)	0.107	1.10 (0.82–1.49)	0.513	1.03 (0.77–1.38)	0.852	0.36 (0.18–0.70)	0.003
30 day infection-related readmission, OR^f^	0.60 (0.42–0.86)	0.006	0.57 (0.39–0.83)	0.003	0.57 (0.39–0.83)	0.003	0.57 (0.20–1.61)	0.290
Treatment outcome^g^								
ȃDeath, csHR							0.74 (0.40–1.37)	0.337
ȃRe-infection, csHR							0.26 (0.09–0.74)	0.011
ȃSuccess, csHR							2.45 (1.59–3.77)	<0.001

All effects are corrected for baseline differences in patient sex, age, ward of hospitalization, and history of previous hospitalization. ES, effect size attributed to the intervention.

Compares the pre-implementation cohort (*n* = 842) with the post-implementation cohort (*n* = 426).

Compares the pre-implementation cohort (*n* = 842) with the post-implementation cohort excluding patients for whom the intervention was not accepted (*n* = 380).

Compares patients who did not receive the intervention in either the pre- or the post-implementation period (*n* = 888) with those who received the intervention (*n* = 380).

Compares patients for whom the intervention was accepted (*n* = 380) with patients for whom the intervention was not accepted (*n* = 46) in the post-implementation period.

Includes death from any cause within 30 days of initiation of carbapenem therapy.

Assessed within 30 days of hospital discharge alive.

Assessed during or at the end of treatment with carbapenems.

Apart from age, demographic and clinical characteristics did not differ substantially between patients with and without acceptance of ASP recommendations during the post-implementation period, but patient outcomes were worse for the latter (Table 3). Multivariable analysis confirmed that patients for whom ASP recommendations were accepted had lower daily hazard of in-hospital death (csHR 0.49; 95% CI 0.30–0.80), lower odds of 30 day mortality (OR 0.36; 95% CI 0.18–0.70) and, albeit not statistically significant, lower odds of infection-related readmission (OR 0.57; 95% CI 0.20–1.61) compared with patients for whom the intervention was not accepted. Moreover, acceptance of the ASP intervention was associated with a higher rate of treatment success (csHR 2.45; 95% CI 1.59–3.77) (Table 4).

## Discussion

This study describes the implementation of a carbapenem-focused ASP during the first year of the COVID-19 pandemic and its impact on the consumption of several broad-spectrum antibiotics with activity against MDR-GNB and on patient outcomes. It is one of the few studies to assess a hospital ASP for carbapenems in Greece, a country with high rates of antibiotic consumption and AMR. The results demonstrate that judicious use of carbapenems in a setting with high rates of MDR-GNB was feasible and led to a significant decrease of their consumption and, importantly, improvement of patient outcomes.

The implementation and maintenance of the ASP in our hospital during the first phases of the COVID-19 pandemic was a challenging and laborious process. At its beginning, the pandemic caused a tremendous depletion of human and structural resources in many hospitals worldwide, compromising their antimicrobial stewardship activities.^15^ This resulted in increased consumption of antimicrobials in hospitals, including carbapenems,^16^ even though medical guidelines regarding the administration of this class of antibiotics had not changed during the COVID-19 pandemic. In some cases, the increased antimicrobial consumption was mitigated by reinstating stewardship activities^17^ or by intensifying ongoing ASPs.^14^ These observations are in accordance with the findings of our study regarding the feasibility of an effective ASP during the COVID-19 period.

The two core strategies of an ASP for inpatient populations include formulary restriction and pre-authorization, and prospective audit and feedback to prescribers. These strategies can be applied separately or in combination. In the pre-pandemic era, both strategies have been shown to effectively and safely reduce unnecessary in-hospital antibiotic use.^18^ However, few relevant data exist during the COVID-19 pandemic. The initial lack of evidence on optimal management of COVID-19 and the accompanying fear of it, the overwhelmed hospitals amid COVID-19 surges, the shortage of available skilled doctors for the implementation or maintenance of ASPs, and medication supply problems, had created an entirely new situation in the hospital sector that may have affected the performance of ASPs. In a recent study from an academic medical centre in the USA, introduction of restriction criteria regarding meropenem use for 2 months during the third year of the pandemic successfully reduced inappropriate meropenem utilization and hospital length of stay, contributing to significant cost savings for the institution.^19^ On the other hand, the results of the present study confirm the preservation of the efficiency of an ASP based on a prospective audit and feedback strategy. Recently, another study showed that the strengthening of an ASP that was already in place, by using a combination of restrictive policies and persuasive techniques, was successful in safely controlling the observed increase of carbapenem consumption during the first wave of COVID-19 pandemic.^14^ Thus, there is evidence to suggest that both core antimicrobial stewardship strategies continue to be safe and effective during the COVID-19 era.

Several reports before the COVID-19 pandemic described reduced carbapenem use without negatively affecting patient outcomes through the implementation of carbapenem-focused ASPs.^20–25^ However, few studies on antimicrobial stewardship were performed in Greece,^26–28^ and only one study examined a carbapenem-focused intervention.^27^ None of these studies addressed the impact of the intervention on the consumption of newer non-carbapenem antibiotics with activity against MDR-GNB. Our study adds new evidence as the sharp and sustained decrease in carbapenem use achieved by the ASP was associated with an increase only in the consumption of piperacillin/tazobactam, which has a lower ecological impact than carbapenems. Moreover, the carbapenem-focused ASP in this study did not significantly affect the consumption of tigecycline and colistin, which are associated with several toxicities and adverse effects, or the consumption of ceftolozane/tazobactam and ceftazidime/avibactam. The latter is important, considering the need to preserve the efficacy of new antibiotics through their judicious use.

Following the ASP implementation in this study, there were no significant changes in all-cause in-hospital mortality and 30 day mortality after carbapenem initiation. On the contrary, the infection-related readmission rate was lower over the post-implementation period. In addition, when the analysis was restricted to the post-implementation cohort, acceptance of the intervention was associated with reduced in-hospital and 30 day mortality after carbapenem initiation, as well as better treatment outcome. These findings are in accordance with the results of the great majority of hospital ASP studies that measured patient outcomes and reported statistically significant reductions or at least non-significant changes in patient mortality and infection-related readmissions.^29–31^

The difference in treatment outcome between cases with accepted and non-accepted intervention in the post-implementation subgroup was probably due to the optimization of diagnostic work-up and antimicrobial treatment through the acceptance of the intervention, reflecting the benefits of ID consultation on this parameter.^32–34^ However, other factors might have acted as potential sources of bias on the estimation of treatment outcome. First, contrary to cases where ASP team recommendation was declined and further consultation was provided only upon request, unsolicited follow-up consultation was given regularly until completion of antimicrobial treatment when the intervention was accepted, thus enhancing the prompt, continuous and efficient handling of possible new-onset complications in these patients. Furthermore, the intervention was not accepted for a number of patients with terminal illness and without proof of concurrent infection for whom the treating physician hesitated to withdraw antibiotics, thus defying the ASP team recommendation.

Previous studies have reported reductions in length of hospital stay after implementing ASPs.^18,30^ However, we found that patients in the post-implementation group experienced longer length of hospital stay, on average, compared with the pre-implementation group. This finding cannot be fully explained. A possible reason could be found in the decreased proportion of admitted patients that received carbapenems during the intervention period, indicating that treating physicians used carbapenems more judiciously then, reserving them only for severe cases, which, however, required longer hospitalization compared with the cases in the pre-intervention period. Unfortunately, we were unable to retrieve more data on the severity of patients’ illness during the pre-implementation period to test this hypothesis.

A key strength of our study is the use of a multidimensional methodology to assess the impact of a carbapenem-focused ASP on antibiotic use and clinical outcomes. Another important feature is the high rate (89%) of acceptance of the intervention by treating physicians. Furthermore, a cross-sectional survey in our hospital near the end of the post-implementation period showed that the ASP described here had positive impact on doctors’ perceptions, attitudes and practices regarding the management of infections due to MDR microorganisms, and 98.5% of respondents wanted the ASP to continue during the COVID-19 pandemic.^35^ Finally, our study can be easily replicated in settings where targeting a specific antibiotic class is needed and ID physicians are available for this purpose.

The present study is not exempt from limitations. Although our segmental regression analysis of interrupted time series is recommended as a powerful tool to assess temporal trends following an intervention,^36,37^ it shares the same limitations as any analysis of observational data. Our analysis examined level and slope changes in the rates of use of carbapenems and other antibiotics following ASP implementation, by accounting for potential confounding effects by seasonality and varying inpatient population size over time. The absence of differences in demographic and clinical characteristics of the patients before and after ASP implementation provides some assurance that our results are unlikely to have been confounded by differences in local epidemiology between the pre-intervention and intervention periods. However, we cannot completely exclude the possibility of residual confounding by unmeasured factors, such as varying frequency and severity of infections with highly resistant bacteria that would require carbapenems. Our sensitivity analyses produced consistent estimates of the relative reduction in the number of patients treated with carbapenems following the ASP under different statistical modelling specifications, but we must note that adjustment for overdispersion resulted in less precise estimates and higher *P* values. We do not view the latter as a major concern as the *P* values remained relatively low (ranging from 0.056 to 0.107) after we inflated the standard errors for overdispersion, and because a separate analysis of hospital volume data of carbapenem consumption confirmed a significant reduction following the ASP implementation. Furthermore, we did not evaluate the impact of the ASP on AMR or the incidence of *Clostridioides difficile* infection, because we considered that the strengthening of infection prevention and control measures due to COVID-19 in the post-implementation period would be an important confounder. In addition, the retrospective nature of the study in the pre-intervention period did not allow the retrieval of reliable data on patient comorbidities during that period. Moreover, paediatric patients were not included in our study. Lastly, this was a single-centre study in a large academic hospital whose capacity was not exceeded during the study period because of the COVID-19 pandemic, thus limiting the generalizability of our results to hospitals of different size and characteristics.

In conclusion, this study demonstrates that implementing and maintaining a carbapenem-focused ASP is feasible, effective and safe in settings with high rates of MDR-GNB, even during the COVID-19 pandemic. The ASP not only effectively reduced the use of carbapenems, but also led to improved patient outcomes, without increasing the consumption of newer antibiotics.

## Supplementary Material

dkad035_Supplementary_Data

## Supplementary data

Tables S1 to S19 are available as Supplementary data at *JAC* Online.

## References

[1] Meletis G . Carbapenem resistance: overview of the problem and future perspectives. Ther Adv Infect Dis 2016; 3: 15–21. 10.1177/2049936115621709 26862399 PMC4735501

[2] Pavez M, Vieira C, de Araujo MR et al Molecular mechanisms of membrane impermeability in clinical isolates of Enterobacteriaceae exposed to imipenem selective pressure. Int J Antimicrob Agents 2016; 48: 78–85. 10.1016/j.ijantimicag.2016.04.016 27256585

[3] Simner PJ, Antar AAR, Hao S et al Antibiotic pressure on the acquisition and loss of antibiotic resistance genes in *Klebsiella pneumoniae*. J Antimicrob Chemother 2018; 73: 1796–803. 10.1093/jac/dky121 29648629 PMC6005101

[4] ECDC . Antimicrobial Consumption in the EU/EEA (ESAC-Net)—Annual Epidemiological Report for 2020. 2021. https://www.ecdc.europa.eu/en/publications-data/surveillance-antimicrobial-consumption-europe-2020.

[5] ECDC . Surveillance of Antimicrobial Resistance in Europe, 2020 Data. 2022. https://www.ecdc.europa.eu/en/publications-data/surveillance-antimicrobial-resistance-europe-2020.

[6] Cassini A, Högberg LD, Plachouras D et al Attributable deaths and disability-adjusted life-years caused by infections with antibiotic-resistant bacteria in the EU and the European Economic Area in 2015: a population-level modelling analysis. Lancet Infect Dis 2019; 19: 56–66. 10.1016/S1473-3099(18)30605-4 30409683 PMC6300481

[7] Lai CC, Chen SY, Ko WC et al Increased antimicrobial resistance during the COVID-19 pandemic. Int J Antimicrob Agents 2021; 57: 106324. 10.1016/j.ijantimicag.2021.106324 33746045 PMC7972869

[8] Spernovasilis NA, Kofteridis DP. COVID-19 and antimicrobial stewardship: what is the interplay? Infect Control Hosp Epidemiol 2021; 42: 378–9. 10.1017/ice.2020.246 32408916 PMC7253763

[9] NIH . Coronavirus Disease 2019 (COVID-19) Treatment Guidelines. 2022. https://www.covid19treatmentguidelines.nih.gov/.

[10] Langford BJ, So M, Raybardhan S et al Antibiotic prescribing in patients with COVID-19: rapid review and meta-analysis. Clin Microbiol Infect 2021; 27: 520–31. 10.1016/j.cmi.2020.12.018 33418017 PMC7785281

[11] Liew Y, Lee WHL, Tan L et al Antimicrobial stewardship programme: a vital resource for hospitals during the global outbreak of coronavirus disease 2019 (COVID-19). Int J Antimicrob Agents 2020; 56: 106145. 10.1016/j.ijantimicag.2020.106145 32860880 PMC7449939

[12] Castro-Lopes A, Correia S, Leal C et al Increase of antimicrobial consumption in a tertiary care hospital during the first phase of the COVID-19 pandemic. Antibiotics (Basel) 2021; 10: 778. 10.3390/antibiotics10070778 34202340 PMC8300755

[13] Grau S, Echeverria-Esnal D, Gómez-Zorrilla S et al Evolution of antimicrobial consumption during the first wave of COVID-19 pandemic. Antibiotics (Basel) 2021; 10: 132. 10.3390/antibiotics10020132 33573070 PMC7911440

[14] AlBahrani S, Almogbel F, Alanazi W et al Carbapenem use correlates with percentage of patients with COVID-19 in intensive care units. Infection 2022; 10.1007/s15010-022-01867-y. PMC9206090 35716341

[15] Tomczyk S, Taylor A, Brown A et al Impact of the COVID-19 pandemic on the surveillance, prevention and control of antimicrobial resistance: a global survey. J Antimicrob Chemother 2021; 76: 3045–58. 10.1093/jac/dkab300 34473285 PMC8499888

[16] Macera M, Onorato L, Calò F et al The impact of the SARS-Cov2 pandemic on a persuasive educational antimicrobial stewardship program in a University Hospital in Southern Italy: a pre-post study. Antibiotics (Basel) 2021; 10: 1405. 10.3390/antibiotics10111405 34827343 PMC8614883

[17] Meschiari M, Onorato L, Bacca E et al Long-term impact of the COVID-19 pandemic on in-hospital antibiotic consumption and antibiotic resistance: a time series analysis (2015–2021). Antibiotics (Basel) 2022; 11: 826. 10.3390/antibiotics11060826 35740232 PMC9219712

[18] Davey P, Marwick CA, Scott CL et al Interventions to improve antibiotic prescribing practices for hospital inpatients. Cochrane Database Syst Rev 2017; 2: CD003543. 10.1002/14651858.CD003543.pub4 28178770 PMC6464541

[19] Wells DA, Johnson AJ, Lukas JG et al Criteria Restricting Inappropriate Meropenem Empiricism (CRIME): a quasi-experimental carbapenem restriction pilot at a large academic medical centre. Int J Antimicrob Agents 2022; 60: 106661. 10.1016/j.ijantimicag.2022.106661 35988667

[20] García-Rodríguez JF, Bardán-García B, Peña-Rodríguez MF et al Meropenem antimicrobial stewardship program: clinical, economic, and antibiotic resistance impact. Eur J Clin Microbiol Infect Dis 2019; 38: 161–70. 10.1007/s10096-018-3408-2 30367313

[21] Faraone A, Poggi A, Cappugi C et al Inappropriate use of carbapenems in an internal medicine ward: impact of a carbapenem-focused antimicrobial stewardship program. Eur J Intern Med 2020; 78: 50–7. 10.1016/j.ejim.2020.03.017 32303455

[22] García-Rodríguez JF, Bardán-García B, Juiz-González PM et al Long-term carbapenems antimicrobial stewardship program. Antibiotics (Basel) 2020; 10: 15. 10.3390/antibiotics10010015 33375237 PMC7823722

[23] López-Viñau T, Peñalva G, García-Martínez L et al Impact of an antimicrobial stewardship program on the incidence of carbapenem resistant Gram-negative bacilli: an interrupted time-series analysis. Antibiotics (Basel) 2021; 10:586. 10.3390/antibiotics10050586 34065645 PMC8190633

[24] Rungsitsathian K, Wacharachaisurapol N, Nakaranurack C et al Acceptance and outcome of interventions in a meropenem de-escalation antimicrobial stewardship program in pediatrics. Pediatr Int 2021; 63: 1458–65. 10.1111/ped.14703 33740838

[25] Kit-Anan W, Boonsathorn S, Anantasit N et al Handshake stewardship reduces carbapenem prescription in a pediatric critical care setting. Pediatr Int 2022; 64: e15227. 10.1111/ped.15227 35912458

[26] Pitiriga V, Kanellopoulos P, Kampos E et al Antimicrobial stewardship program in a Greek hospital: implementing a mandatory prescription form and prospective audits. Future Microbiol 2018; 13: 889–96. 10.2217/fmb-2018-0020 29661029

[27] Makina A-A, Poulakou G, Sympardi S et al Safety of a carbapenem-sparing approach as part of an antibiotic stewardship program, in a setting with increased carbapenem resistance. Infect Dis Clin Microbiol 2019; 1: 14–25. 10.5152/idcm.2019.19003

[28] Chrysou K, Zarkotou O, Kalofolia S et al Impact of a 4-year antimicrobial stewardship program implemented in a Greek tertiary hospital. Eur J Clin Microbiol Infect Dis 2022; 41: 127–32. 10.1007/s10096-021-04290-7 34264401

[29] Schuts EC, Hulscher M, Mouton JW et al Current evidence on hospital antimicrobial stewardship objectives: a systematic review and meta-analysis. Lancet Infect Dis 2016; 16: 847–56. 10.1016/S1473-3099(16)00065-7 26947617

[30] Nathwani D, Varghese D, Stephens J et al Value of hospital antimicrobial stewardship programs [ASPs]: a systematic review. Antimicrob Resist Infect Control 2019; 8: 35. 10.1186/s13756-019-0471-0 30805182 PMC6373132

[31] Carrara E, Sibani M, Barbato L et al How to ‘SAVE’ antibiotics: effectiveness and sustainability of a new model of antibiotic stewardship intervention in the internal medicine area. Int J Antimicrob Agents 2022; 60: 106672. 10.1016/j.ijantimicag.2022.106672 36103917

[32] Kim SH, Huh K, Cho SY et al Factors associated with the recurrence of acute pyelonephritis caused by extended-spectrum β-lactamase-producing *Escherichia coli*: the importance of infectious disease consultation. Diagn Microbiol Infect Dis 2019; 94: 55–9. 10.1016/j.diagmicrobio.2018.11.019 30642718

[33] Whittington KJ, Ma Y, Butler AM et al The impact of infectious diseases consultation for children with *Staphylococcus aureus* bacteremia. Pediatr Res 2022; 92: 1598–605. 10.1038/s41390-022-02251-0 35982140 PMC9789160

[34] Mejia-Chew C, O’Halloran JA, Olsen MA et al Effect of infectious disease consultation on mortality and treatment of patients with *Candida* bloodstream infections: a retrospective, cohort study. Lancet Infect Dis 2019; 19: 1336–44. 10.1016/S1473-3099(19)30405-0 31562024 PMC7014922

[35] Spernovasilis N, Ierodiakonou D, Spanias C et al Doctors’ perceptions, attitudes and practices towards the management of multidrug-resistant organism infections after the implementation of an antimicrobial stewardship programme during the COVID-19 pandemic. Trop Med Infect Dis 2021; 6: 20. 10.3390/tropicalmed6010020 33562723 PMC7930958

[36] Stone SP, Cooper BS, Kibbler CC et al The ORION statement: guidelines for transparent reporting of outbreak reports and intervention studies of nosocomial infection. J Antimicrob Chemother 2007; 59: 833–40. 10.1093/jac/dkm055 17387116

[37] Schweizer ML, Braun BI, Milstone AM. Research methods in healthcare epidemiology and antimicrobial stewardship-quasi-experimental designs. Infect Control Hosp Epidemiol 2016; 37: 1135–40. 10.1017/ice.2016.117 27267457 PMC5036994

