# Supplementary material for: A carbapenem-focused antimicrobial stewardship programme implemented during the COVID-19 pandemic in a setting of high endemicity for multidrug-resistant Gram-negative bacteria
Source: J Antimicrob Chemother. 2023 Feb 15;78(4):1000–8. doi: 10.1093/jac/dkad035 (PMC11023244; doi:10.1093/jac/dkad035)
Supplement: dkad035_Supplementary_Data [file dkad035_supplementary_data.docx]

Supplementary Statistical Tables

**Table S1.** Results of multivariable Cox proportional hazards regression quantifying the effects of the antimicrobial stewardship intervention on all-cause hospital mortality. This is an ITT analysis comparing the pre-implementation cohort (n=842) to the post-implementation cohort (n=426).

|  | csHR | Std. error | 95% CI | Wald Z | p-value |
| --- | --- | --- | --- | --- | --- |
| ITT analysis |  |  |  |  |  |
| Pre- implementation group | 1.00 | 0.00 |  |  |  |
| Post- implementation group | 0.99 | 0.12 | [0.79, 1.24] | -0.10 | 0.922 |
| Sex |  |  |  |  |  |
| Female | 1.00 | 0.00 |  |  |  |
| Male | 0.97 | 0.11 | [0.77, 1.22] | -0.25 | 0.805 |
| Age (years) | 1.04 | 0.00 | [1.03, 1.05] | 9.04 | <0.001 |
| Ward of hospitalization |  |  |  |  |  |
| Intensive care | 2.71 | 0.53 | [1.85, 3.97] | 5.14 | <0.001 |
| Medicine | 2.17 | 0.38 | [1.53, 3.07] | 4.38 | <0.001 |
| Surgery | 1.00 | 0.00 |  |  |  |
| Other specialty | 5.23 | 5.33 | [0.71, 38.59] | 1.62 | 0.104 |
| Hospitalization in previous 3 months |  |  |  |  |  |
| No | 1.00 | 0.00 |  |  |  |
| Yes | 1.61 | 0.19 | [1.28, 2.04] | 4.04 | <0.001 |

Abbreviations: csHR, cause-specific hazard ratio

**Table S2.** Results of multivariable Cox proportional hazards regression quantifying the effects of the antimicrobial stewardship intervention on hospital discharge alive. This is an ITT analysis comparing the pre-implementation cohort (n=842) to the post-implementation cohort (n=426).

|  | csHR | Std. error | 95% CI | Wald Z | p-value |
| --- | --- | --- | --- | --- | --- |
| ITT analysis |  |  |  |  |  |
| Pre-implementation group | 1.00 | 0.00 |  |  |  |
| Post-implementation group | 0.80 | 0.06 | [0.70, 0.92] | -3.16 | 0.002 |
| Sex |  |  |  |  |  |
| Female | 1.00 | 0.00 |  |  |  |
| Male | 0.97 | 0.07 | [0.85, 1.11] | -0.40 | 0.690 |
| Age (years) | 1.00 | 0.00 | [1.00, 1.01] | 1.48 | 0.139 |
| Ward of hospitalization |  |  |  |  |  |
| Intensive care | 0.33 | 0.05 | [0.25, 0.43] | -8.05 | <0.001 |
| Medicine | 0.96 | 0.07 | [0.83, 1.12] | -0.48 | 0.630 |
| Surgery | 1.00 | 0.00 |  |  |  |
| Other specialty | 2.90 | 1.12 | [1.36, 6.19] | 2.76 | 0.006 |
| Hospitalization in previous 3 months |  |  |  |  |  |
| No | 1.00 | 0.00 |  |  |  |
| Yes | 1.16 | 0.08 | [1.02, 1.33] | 2.27 | 0.023 |

Abbreviations: csHR, cause-specific hazard ratio

Note. In this analysis, a low csHR for discharge alive reflects a low daily rate of discharge, resulting in prolonged hospital stay.

**Table S3.** Results of multivariable Logistic regression quantifying the effects of the antimicrobial stewardship intervention on all-cause mortality within 30 days of initiation of carbapenem therapy. This is an ITT analysis comparing the pre-implementation cohort (n=842) to the post-implementation cohort (n=426).

|  | OR | Std. error | 95% CI | Wald Z | p-value |
| --- | --- | --- | --- | --- | --- |
| ITT analysis |  |  |  |  |  |
| Pre-implementation group | 1.00 | 0.00 |  |  |  |
| Post-implementation group | 1.26 | 0.18 | [0.95, 1.67] | 1.61 | 0.107 |
| Sex |  |  |  |  |  |
| Female | 1.00 | 0.00 |  |  |  |
| Male | 1.06 | 0.15 | [0.80, 1.41] | 0.42 | 0.672 |
| Age (years) | 1.03 | 0.01 | [1.02, 1.04] | 6.61 | <0.001 |
| Ward of hospitalization |  |  |  |  |  |
| Intensive care | 9.34 | 2.29 | [5.78, 15.09] | 9.12 | <0.001 |
| Medicine | 2.04 | 0.39 | [1.39, 2.97] | 3.68 | <0.001 |
| Surgery | 1.00 | 0.00 |  |  |  |
| Other specialty | 1.50 | 1.66 | [0.17, 13.05] | 0.37 | 0.714 |
| Hospitalization in previous 3 months |  |  |  |  |  |
| No | 1.00 | 0.00 |  |  |  |
| Yes | 1.22 | 0.18 | [0.93, 1.62] | 1.42 | 0.156 |
| Intercept | 0.01 | 0.01 | [0.01, 0.03] | -10.65 | <0.001 |

**Table S4.** Results of multivariable Logistic regression quantifying the effects of the antimicrobial stewardship intervention on infection-related readmission within 30 days of hospital discharge alive. This is an ITT analysis comparing the pre-implementation cohort (n=842) to the post-implementation cohort (n=426).

|  | OR | Std. error | 95% CI | Wald Z | p-value |
| --- | --- | --- | --- | --- | --- |
| ITT analysis |  |  |  |  |  |
| Pre-implementation group | 1.00 | 0.00 |  |  |  |
| Post-implementation group | 0.60 | 0.11 | [0.42, 0.86] | -2.76 | 0.006 |
| Sex |  |  |  |  |  |
| Female | 1.00 | 0.00 |  |  |  |
| Male | 1.44 | 0.25 | [1.02, 2.03] | 2.09 | 0.037 |
| Age (years) | 1.01 | 0.01 | [1.00, 1.02] | 1.40 | 0.163 |
| Ward of hospitalization |  |  |  |  |  |
| Intensive care | 1.16 | 0.47 | [0.52, 2.57] | 0.36 | 0.716 |
| Medicine | 2.04 | 0.41 | [1.37, 3.02] | 3.53 | <0.001 |
| Surgery | 1.00 | 0.00 |  |  |  |
| Other specialty | 1.35 | 1.51 | [0.15, 12.04] | 0.27 | 0.786 |
| Hospitalization in previous 3 months |  |  |  |  |  |
| No | 1.00 | 0.00 |  |  |  |
| Yes | 1.82 | 0.32 | [1.30, 2.56] | 3.46 | 0.001 |
| Intercept | 0.07 | 0.03 | [0.03, 0.15] | -6.64 | <0.001 |

**Table S5.** Results of multivariable Cox proportional hazards regression quantifying the effects of the antimicrobial stewardship intervention on all-cause hospital mortality. This is a modified ITT analysis comparing the pre-implementation cohort (n=842) to the post-implementation cohort excluding patients for whom the intervention was not accepted (n=380).

|  | csHR | Std. error | 95% CI | Wald Z | p-value |
| --- | --- | --- | --- | --- | --- |
| Modified ITT analysis |  |  |  |  |  |
| Pre-implementation group | 1.00 | 0.00 |  |  |  |
| Post-implementation intervention-accepted  group | 0.91 | 0.11 | [0.71, 1.16] | -0.76 | 0.450 |
| Sex |  |  |  |  |  |
| Female | 1.00 | 0.00 |  |  |  |
| Male | 0.95 | 0.12 | [0.75, 1.20] | -0.45 | 0.652 |
| Age (years) | 1.04 | 0.00 | [1.03, 1.05] | 8.81 | <0.001 |
| Ward of hospitalization |  |  |  |  |  |
| Intensive care | 2.85 | 0.58 | [1.91, 4.25] | 5.13 | <0.001 |
| Medicine | 2.26 | 0.42 | [1.56, 3.27] | 4.34 | <0.001 |
| Surgery | 1.00 | 0.00 |  |  |  |
| Other specialty | 5.64 | 5.77 | [0.76, 41.80] | 1.69 | 0.090 |
| Hospitalization in previous 3 months |  |  |  |  |  |
| No | 1.00 | 0.00 |  |  |  |
| Yes | 1.65 | 0.20 | [1.29, 2.10] | 4.04 | <0.001 |

Abbreviations: csHR, cause-specific hazard ratio

**Table S6.** Results of multivariable Cox proportional hazards regression quantifying the effects of the antimicrobial stewardship intervention on hospital discharge alive. This is a modified ITT analysis comparing the pre-implementation cohort (n=842) to the post-implementation cohort excluding patients for whom the intervention was not accepted (n=380).

|  | csHR | Std. error | 95% CI | Wald Z | p-value |
| --- | --- | --- | --- | --- | --- |
| Modified ITT analysis |  |  |  |  |  |
| Pre-implementation group | 1.00 | 0.00 |  |  |  |
| Post-implementation intervention-accepted  group | 0.83 | 0.06 | [0.72, 0.95] | -2.62 | 0.009 |
| Sex |  |  |  |  |  |
| Female | 1.00 | 0.00 |  |  |  |
| Male | 0.97 | 0.07 | [0.84, 1.11] | -0.50 | 0.616 |
| Age (years) | 1.00 | 0.00 | [1.00, 1.01] | 1.78 | 0.075 |
| Ward of hospitalization |  |  |  |  |  |
| Intensive care | 0.33 | 0.05 | [0.25, 0.43] | -8.09 | <0.001 |
| Medicine | 0.96 | 0.07 | [0.83, 1.12] | -0.50 | 0.614 |
| Surgery | 1.00 | 0.00 |  |  |  |
| Other specialty | 2.88 | 1.11 | [1.35, 6.13] | 2.74 | 0.006 |
| Hospitalization in previous 3 months |  |  |  |  |  |
| No | 1.00 | 0.00 |  |  |  |
| Yes | 1.17 | 0.08 | [1.02, 1.34] | 2.31 | 0.021 |

Abbreviations: csHR, cause-specific hazard ratio

Note. In this analysis, a low csHR for discharge alive reflects a low daily rate of discharge, resulting in prolonged hospital stay.

**Table S7.** Results of multivariable Logistic regression quantifying the effects of the antimicrobial stewardship intervention on all-cause mortality within 30 days of initiation of carbapenem therapy. This is a modified ITT analysis comparing the pre-implementation cohort (n=842) to the post-implementation cohort excluding patients for whom the intervention was not accepted (n=380).

|  | OR | Std. error | 95% CI | Wald Z | p-value |
| --- | --- | --- | --- | --- | --- |
| Modified ITT analysis |  |  |  |  |  |
| Pre-intervention group | 1.00 | 0.00 |  |  |  |
| Post-implementation intervention-  accepted group | 1.10 | 0.17 | [0.82, 1.49] | 0.65 | 0.513 |
| Sex |  |  |  |  |  |
| Female | 1.00 | 0.00 |  |  |  |
| Male | 1.06 | 0.16 | [0.79, 1.42] | 0.40 | 0.691 |
| Age (years) | 1.03 | 0.01 | [1.02, 1.04] | 6.35 | <0.001 |
| Ward of hospitalization |  |  |  |  |  |
| Intensive care | 10.00 | 2.53 | [6.09, 16.43] | 9.10 | <0.001 |
| Medicine | 2.13 | 0.43 | [1.43, 3.18] | 3.73 | <0.001 |
| Surgery | 1.00 | 0.00 |  |  |  |
| Other specialty | 1.68 | 1.86 | [0.19, 14.66] | 0.47 | 0.636 |
| Hospitalization in previous 3 months |  |  |  |  |  |
| No | 1.00 | 0.00 |  |  |  |
| Yes | 1.25 | 0.18 | [0.94, 1.67] | 1.51 | 0.130 |
| Intercept | 0.01 | 0.01 | [0.01, 0.03] | -10.37 | <0.001 |

**Table S8.** Results of multivariable Logistic regression quantifying the effects of the antimicrobial stewardship intervention on infection-related readmission within 30 days of hospital discharge alive. This is a modified ITT analysis comparing the pre-implementation cohort (n=842) to the post-implementation cohort excluding patients for whom the intervention was not accepted (n=380).

|  | OR | Std. error | 95% CI | Wald Z | p-value |
| --- | --- | --- | --- | --- | --- |
| Modified ITT analysis |  |  |  |  |  |
| Pre-implementation group | 1.00 | 0.00 |  |  |  |
| Post-implementation intervention-  accepted group | 0.57 | 0.11 | [0.39, 0.83] | -2.93 | 0.003 |
| Sex |  |  |  |  |  |
| Female | 1.00 | 0.00 |  |  |  |
| Male | 1.37 | 0.24 | [0.97, 1.94] | 1.78 | 0.075 |
| Age (years) | 1.01 | 0.01 | [1.00, 1.02] | 1.24 | 0.216 |
| Ward of hospitalization |  |  |  |  |  |
| Intensive care | 1.16 | 0.47 | [0.52, 2.57] | 0.36 | 0.720 |
| Medicine | 1.99 | 0.41 | [1.33, 2.97] | 3.37 | 0.001 |
| Surgery | 1.00 | 0.00 |  |  |  |
| Other specialty | 1.31 | 1.46 | [0.15, 11.65] | 0.24 | 0.809 |
| Hospitalization in previous 3 months |  |  |  |  |  |
| No | 1.00 | 0.00 |  |  |  |
| Yes | 1.80 | 0.32 | [1.27, 2.54] | 3.34 | 0.001 |
| Intercept | 0.08 | 0.03 | [0.03, 0.17] | -6.32 | <0.001 |

**Table S9.** Results of multivariable Cox proportional hazards regression quantifying the effects of the antimicrobial stewardship intervention on all-cause hospital mortality. This is an analysis per protocol, comparing patients who did not receive the intervention in either the pre- or the post-implementation period (n=888) to those who received the intervention (n=380).

|  | csHR | Std. error | 95% CI | Wald Z | p-value |
| --- | --- | --- | --- | --- | --- |
| Per-protocol analysis |  |  |  |  |  |
| Non-intervention group | 1.00 | 0.00 |  |  |  |
| Intervention group | 0.87 | 0.11 | [0.69, 1.11] | -1.14 | 0.256 |
| Sex |  |  |  |  |  |
| Female | 1.00 | 0.00 |  |  |  |
| Male | 0.97 | 0.11 | [0.77, 1.22] | -0.28 | 0.779 |
| Age (years) | 1.04 | 0.00 | [1.03, 1.05] | 9.09 | <0.001 |
| Ward of hospitalization |  |  |  |  |  |
| Intensive care | 2.74 | 0.53 | [1.87, 4.00] | 5.18 | <0.001 |
| Medicine | 2.17 | 0.38 | [1.53, 3.06] | 4.38 | <0.001 |
| Surgery | 1.00 | 0.00 |  |  |  |
| Other specialty | 5.47 | 5.58 | [0.74, 40.39] | 1.67 | 0.095 |
| Hospitalization in previous 3 months |  |  |  |  |  |
| No | 1.00 | 0.00 |  |  |  |
| Yes | 1.59 | 0.19 | [1.26, 2.01] | 3.93 | <0.001 |

Abbreviations: csHR, cause-specific hazard ratio

**Table S10.** Results of multivariable Cox proportional hazards regression quantifying the effects of the antimicrobial stewardship intervention on hospital discharge alive. This is an analysis per protocol, comparing patients who did not receive the intervention in either the pre- or the post-implementation period (n=888) to those who received the intervention (n=380).

|  | csHR | Std. error | 95% CI | Wald Z | p-value |
| --- | --- | --- | --- | --- | --- |
| Per-protocol analysis |  |  |  |  |  |
| Non-intervention group | 1.00 | 0.00 |  |  |  |
| Intervention group | 0.85 | 0.06 | [0.74, 0.98] | -2.29 | 0.022 |
| Sex |  |  |  |  |  |
| Female | 1.00 | 0.00 |  |  |  |
| Male | 0.97 | 0.07 | [0.85, 1.11] | -0.40 | 0.692 |
| Age (years) | 1.00 | 0.00 | [1.00, 1.01] | 1.35 | 0.178 |
| Ward of hospitalization |  |  |  |  |  |
| Intensive care | 0.33 | 0.05 | [0.25, 0.44] | -7.99 | <0.001 |
| Medicine | 0.97 | 0.07 | [0.84, 1.12] | -0.44 | 0.659 |
| Surgery | 1.00 | 0.00 |  |  |  |
| Other specialty | 2.91 | 1.12 | [1.36, 6.19] | 2.77 | 0.006 |
| Hospitalization in previous 3 months |  |  |  |  |  |
| No | 1.00 | 0.00 |  |  |  |
| Yes | 1.17 | 0.08 | [1.03, 1.34] | 2.36 | 0.018 |

Abbreviations: csHR, cause-specific hazard ratio

Note. In this analysis, a low csHR for discharge alive reflects a low daily rate of discharge, resulting in prolonged hospital stay.

**Table S11.** Results of multivariable Logistic regression quantifying the effects of the antimicrobial stewardship intervention on all-cause mortality within 30 days of initiation of carbapenem therapy. This is an analysis per protocol, comparing patients who did not receive the intervention in either the pre- or the post-implementation period (n=888) to those who received the intervention (n=380).

|  | OR | Std. error | 95% CI | Wald Z | p-value |
| --- | --- | --- | --- | --- | --- |
| Per-protocol analysis |  |  |  |  |  |
| Non-intervention group | 1.00 | 0.00 |  |  |  |
| Intervention group | 1.03 | 0.15 | [0.77, 1.38] | 0.19 | 0.852 |
| Sex |  |  |  |  |  |
| Female | 1.00 | 0.00 |  |  |  |
| Male | 1.06 | 0.15 | [0.80, 1.41] | 0.40 | 0.686 |
| Age (years) | 1.03 | 0.00 | [1.02, 1.04] | 6.65 | <0.001 |
| Ward of hospitalization |  |  |  |  |  |
| Intensive care | 9.38 | 2.30 | [5.80, 15.16] | 9.14 | <0.001 |
| Medicine | 2.04 | 0.39 | [1.40, 2.98] | 3.70 | <0.001 |
| Surgery | 1.00 | 0.00 |  |  |  |
| Other specialty | 1.59 | 1.74 | [0.18, 13.70] | 0.42 | 0.676 |
| Hospitalization in previous 3 months |  |  |  |  |  |
| No | 1.00 | 0.00 |  |  |  |
| Yes | 1.21 | 0.17 | [0.92, 1.61] | 1.36 | 0.174 |
| Intercept | 0.01 | 0.01 | [0.01, 0.03] | -10.51 | <0.001 |

**Table S12.** Results of multivariable Logistic regression quantifying the effects of the antimicrobial stewardship intervention on infection-related readmission within 30 days of hospital discharge alive. This is an analysis per protocol, comparing patients who did not receive the intervention in either the pre- or the post-implementation period (n=888) to those who received the intervention (n=380).

|  | OR | Std. error | 95% CI | Wald Z | p-value |
| --- | --- | --- | --- | --- | --- |
| Per-protocol analysis |  |  |  |  |  |
| Non-intervention group | 1.00 | 0.00 |  |  |  |
| Intervention group | 0.57 | 0.11 | [0.39, 0.83] | -2.96 | 0.003 |
| Sex |  |  |  |  |  |
| Female | 1.00 | 0.00 |  |  |  |
| Male | 1.43 | 0.25 | [1.02, 2.02] | 2.05 | 0.040 |
| Age (years) | 1.01 | 0.01 | [1.00, 1.02] | 1.37 | 0.172 |
| Ward of hospitalization |  |  |  |  |  |
| Intensive care | 1.19 | 0.48 | [0.53, 2.63] | 0.42 | 0.674 |
| Medicine | 2.05 | 0.41 | [1.38, 3.04] | 3.56 | <0.001 |
| Surgery | 1.00 | 0.00 |  |  |  |
| Other specialty | 1.38 | 1.54 | [0.15, 12.29] | 0.29 | 0.774 |
| Hospitalization in previous 3 months |  |  |  |  |  |
| No | 1.00 | 0.00 |  |  |  |
| Yes | 1.84 | 0.32 | [1.31, 2.59] | 3.52 | <0.001 |
| Intercept | 0.07 | 0.03 | [0.03, 0.15] | -6.63 | <0.001 |

**Table S13.** Results of multivariable Cox proportional hazards regression quantifying the effects of the antimicrobial stewardship intervention on all-cause hospital mortality. This analysis is restricted in the post-implementation period and compares patients for whom the intervention was accepted (n=380) to patients for whom the intervention was not accepted (n=46).

|  | csHR | Std. error | 95% CI | Wald Z | p-value |
| --- | --- | --- | --- | --- | --- |
| Intervention acceptance |  |  |  |  |  |
| Intervention non-accepted group | 1.00 | 0.00 |  |  |  |
| Intervention accepted group | 0.49 | 0.12 | [0.30, 0.80] | -2.85 | 0.004 |
| Sex |  |  |  |  |  |
| Female | 1.00 | 0.00 |  |  |  |
| Male | 0.98 | 0.19 | [0.66, 1.43] | -0.13 | 0.898 |
| Age (years) | 1.03 | 0.01 | [1.02, 1.05] | 4.72 | <0.001 |
| Ward of hospitalization |  |  |  |  |  |
| Intensive care | 2.97 | 0.96 | [1.57, 5.60] | 3.36 | 0.001 |
| Medicine | 2.35 | 0.68 | [1.34, 4.15] | 2.96 | 0.003 |
| Surgery | 1.00 | 0.00 |  |  |  |
| Other specialty | 9.04 | 9.53 | [1.15, 71.28] | 2.09 | 0.037 |
| Hospitalization in previous 3 months |  |  |  |  |  |
| No | 1.00 | 0.00 |  |  |  |
| Yes | 1.99 | 0.40 | [1.34, 2.95] | 3.41 | 0.001 |

Abbreviations: csHR, cause-specific hazard ratio

**Table S14.** Results of multivariable Cox proportional hazards regression quantifying the effects of the antimicrobial stewardship intervention on hospital discharge alive. This analysis is restricted in the post-implementation period and compares patients for whom the intervention was accepted (n=380) to patients for whom the intervention was not accepted (n=46).

|  | csHR | Std. error | 95% CI | Wald Z | p-value |
| --- | --- | --- | --- | --- | --- |
| Intervention acceptance |  |  |  |  |  |
| Intervention non-accepted group | 1.00 | 0.00 |  |  |  |
| Intervention accepted group | 1.32 | 0.28 | [0.87, 2.00] | 1.32 | 0.187 |
| Sex |  |  |  |  |  |
| Female | 1.00 | 0.00 |  |  |  |
| Male | 1.04 | 0.13 | [0.82, 1.33] | 0.36 | 0.718 |
| Age (years) | 1.00 | 0.00 | [0.99, 1.01] | -0.07 | 0.944 |
| Ward of hospitalization |  |  |  |  |  |
| Intensive care | 0.40 | 0.09 | [0.26, 0.63] | -3.94 | <0.001 |
| Medicine | 1.08 | 0.15 | [0.83, 1.42] | 0.58 | 0.562 |
| Surgery | 1.00 | 0.00 |  |  |  |
| Other specialty | 3.52 | 2.11 | [1.09, 11.39] | 2.10 | 0.036 |
| Hospitalization in previous 3 months |  |  |  |  |  |
| No | 1.00 | 0.00 |  |  |  |
| Yes | 1.37 | 0.17 | [1.08, 1.74] | 2.62 | 0.009 |

Abbreviations: csHR, cause-specific hazard ratio

Note. In this analysis, a low csHR for discharge alive reflects a low daily rate of discharge, resulting in prolonged hospital stay.

**Table S15.** Results of multivariable Logistic regression quantifying the effects of the antimicrobial stewardship intervention on all-cause mortality within 30 days of initiation of carbapenem therapy. This analysis is restricted in the post-implementation period and compares patients for whom the intervention was accepted (n=380) to patients for whom the intervention was not accepted (n=46).

|  | OR | Std. error | 95% CI | Wald Z | p-value |
| --- | --- | --- | --- | --- | --- |
| Intervention acceptance |  |  |  |  |  |
| Intervention non-accepted group | 1.00 | 0.00 |  |  |  |
| Intervention accepted group | 0.36 | 0.12 | [0.18, 0.70] | -3.02 | 0.003 |
| Sex |  |  |  |  |  |
| Female | 1.00 | 0.00 |  |  |  |
| Male | 0.94 | 0.22 | [0.59, 1.49] | -0.27 | 0.786 |
| Age (years) | 1.03 | 0.01 | [1.01, 1.04] | 3.37 | 0.001 |
| Ward of hospitalization |  |  |  |  |  |
| Intensive care | 6.54 | 2.59 | [3.01, 14.19] | 4.75 | <0.001 |
| Medicine | 1.66 | 0.51 | [0.90, 3.04] | 1.63 | 0.103 |
| Surgery | 1.00 | 0.00 |  |  |  |
| Other specialty | 2.22 | 2.69 | [0.21, 23.80] | 0.66 | 0.511 |
| Hospitalization in previous 3 months |  |  |  |  |  |
| No | 1.00 | 0.00 |  |  |  |
| Yes | 1.19 | 0.28 | [0.75, 1.89] | 0.73 | 0.462 |
| Intercept | 0.09 | 0.06 | [0.02, 0.33] | -3.56 | <0.001 |

**Table S16.** Results of multivariable Logistic regression quantifying the effects of the antimicrobial stewardship intervention on infection-related readmission within 30 days of hospital discharge alive. This analysis is restricted in the post-implementation period and compares patients for whom the intervention was accepted (n=380) to patients for whom the intervention was not accepted (n=46).

|  | OR | Std. error | 95% CI | Wald Z | p-value |
| --- | --- | --- | --- | --- | --- |
| Intervention acceptance |  |  |  |  |  |
| Intervention non-accepted group | 1.00 | 0.00 |  |  |  |
| Intervention accepted group | 0.57 | 0.30 | [0.20, 1.61] | -1.06 | 0.290 |
| Sex |  |  |  |  |  |
| Female | 1.00 | 0.00 |  |  |  |
| Male | 1.78 | 0.63 | [0.89, 3.56] | 1.64 | 0.102 |
| Age (years) | 1.02 | 0.01 | [1.00, 1.05] | 2.06 | 0.039 |
| Ward of hospitalization |  |  |  |  |  |
| Intensive care | 1.34 | 0.98 | [0.32, 5.62] | 0.40 | 0.689 |
| Medicine | 1.92 | 0.79 | [0.86, 4.31] | 1.58 | 0.113 |
| Surgery | 1.00 | 0.00 |  |  |  |
| Other specialty | 1.00 | 0.00 |  |  |  |
| Hospitalization in previous 3 months |  |  |  |  |  |
| No | 1.00 | 0.00 |  |  |  |
| Yes | 2.09 | 0.72 | [1.06, 4.11] | 2.13 | 0.033 |
| Intercept | 0.02 | 0.02 | [0.00, 0.14] | -3.88 | <0.001 |

**Table S17.** Results of multivariable Cox proportional hazards regression quantifying the effects of the antimicrobial stewardship intervention on all-cause hospital mortality. This analysis is restricted in the post-implementation period and compares patients for whom the intervention was accepted (n=380) to patients for whom the intervention was not accepted (n=46).

|  | csHR | Std. error | 95% CI | Wald Z | p-value |
| --- | --- | --- | --- | --- | --- |
| Intervention acceptance |  |  |  |  |  |
| Intervention non-accepted group | 1.00 | 0.00 |  |  |  |
| Intervention accepted group | 0.74 | 0.23 | [0.40, 1.37] | -0.96 | 0.337 |
| Sex |  |  |  |  |  |
| Female | 1.00 | 0.00 |  |  |  |
| Male | 0.97 | 0.23 | [0.61, 1.55] | -0.12 | 0.906 |
| Age (years) | 1.02 | 0.01 | [1.01, 1.04] | 2.62 | 0.009 |
| Ward of hospitalization |  |  |  |  |  |
| Intensive care | 3.18 | 1.36 | [1.37, 7.37] | 2.70 | 0.007 |
| Medicine | 1.69 | 0.62 | [0.82, 3.49] | 1.42 | 0.155 |
| Surgery | 1.00 | 0.00 |  |  |  |
| Other specialty | 2.90 | 3.11 | [0.35, 23.70] | 0.99 | 0.321 |
| Hospitalization in previous 3 months |  |  |  |  |  |
| No | 1.00 | 0.00 |  |  |  |
| Yes | 1.46 | 0.36 | [0.90, 2.37] | 1.52 | 0.127 |

Abbreviations: csHR, cause-specific hazard ratio

**Table S18.** Results of multivariable Cox proportional hazards regression quantifying the effects of the antimicrobial stewardship intervention on new or recurrent infection. This analysis is restricted in the post-implementation period and compares patients for whom the intervention was accepted (n=380) to patients for whom the intervention was not accepted (n=46).

|  | csHR | Std. error | 95% CI | Wald Z | p-value |
| --- | --- | --- | --- | --- | --- |
| Intervention acceptance |  |  |  |  |  |
| Intervention non-accepted group | 1.00 | 0.00 |  |  |  |
| Intervention accepted group | 0.26 | 0.14 | [0.09, 0.74] | -2.53 | 0.011 |
| Sex |  |  |  |  |  |
| Female | 1.00 | 0.00 |  |  |  |
| Male | 1.02 | 0.55 | [0.36, 2.92] | 0.04 | 0.966 |
| Age (years) | 1.02 | 0.02 | [0.99, 1.05] | 1.09 | 0.275 |
| Ward of hospitalization |  |  |  |  |  |
| Intensive care | 0.88 | 0.78 | [0.15, 4.98] | -0.15 | 0.881 |
| Medicine | 0.69 | 0.39 | [0.22, 2.11] | -0.66 | 0.512 |
| Surgery | 1.00 | 0.00 |  |  |  |
| Other specialty | 0.00 | 0.00 | [0.00, .] | -0.00 | >0.999 |
| Hospitalization in previous 3 months |  |  |  |  |  |
| No | 1.00 | 0.00 |  |  |  |
| Yes | 0.42 | 0.22 | [0.14, 1.20] | -1.63 | 0.103 |

Abbreviations: csHR, cause-specific hazard ratio

**Table S19.** Results of multivariable Cox proportional hazards regression quantifying the effects of the antimicrobial stewardship intervention on favourable treatment outcome. This analysis is restricted in the post-implementation period and compares patients for whom the intervention was accepted (n=380) to patients for whom the intervention was not accepted (n=46).

|  | csHR | Std. error | 95% CI | Wald Z | p-value |
| --- | --- | --- | --- | --- | --- |
| Intervention acceptance |  |  |  |  |  |
| Intervention non-accepted group | 1.00 | 0.00 |  |  |  |
| Intervention accepted group | 2.45 | 0.54 | [1.59, 3.77] | 4.09 | <0.001 |
| Sex |  |  |  |  |  |
| Female | 1.00 | 0.00 |  |  |  |
| Male | 1.00 | 0.12 | [0.80, 1.26] | 0.04 | 0.969 |
| Age (years) | 1.00 | 0.00 | [0.99, 1.00] | -1.35 | 0.177 |
| Ward of hospitalization |  |  |  |  |  |
| Intensive care | 0.60 | 0.12 | [0.41, 0.89] | -2.53 | 0.012 |
| Medicine | 0.86 | 0.11 | [0.66, 1.12] | -1.14 | 0.255 |
| Surgery | 1.00 | 0.00 |  |  |  |
| Other specialty | 0.81 | 0.48 | [0.25, 2.61] | -0.35 | 0.728 |
| Hospitalization in previous 3 months |  |  |  |  |  |
| No | 1.00 | 0.00 |  |  |  |
| Yes | 0.88 | 0.10 | [0.70, 1.10] | -1.14 | 0.254 |

Abbreviations: csHR, cause-specific hazard ratio
